# *Kueselia aquadivae* gen. nov., sp. nov., the first member of the family *Isosphaeraceae* isolated from subsurface percolates

**DOI:** 10.1038/s41598-025-17081-3

**Published:** 2025-09-01

**Authors:** Madeleine Kündgen, Tom Haufschild, Jonathan Hammer, Katharina Lehmann, Madeleine Mutter, Mareike Jogler, He Wang, Robert Lehmann, Nicolai Kallscheuer, Kai Uwe Totsche, Christian Jogler

**Affiliations:** 1 Department of Microbial Interactions, Institute of Microbiology, Friedrich Schiller University (FSU), Jena, Germany; 2 Department of Hydrogeology, Institute of Geosciences, Friedrich Schiller University (FSU), Jena, Germany; 3 Aquatic Geomicrobiology, Institute of Biodiversity, Friedrich Schiller University (FSU), Jena, Germany; 4 Cluster of Excellence Balance of the Microverse, Friedrich Schiller University Jena (FSU), Jena, Germany

**Keywords:** Critical zone, Aeration zone, Drainage collectors, Geomicrobiology, *Isosphaeraceae*, *Planctomycetota*, Biogeochemistry, Freshwater ecology

## Abstract

Subsurface habitats, found under various geological conditions, exhibit diverse microbial communities. The vadose zone, a previously unexplored subsurface compartment, connects the surface to phreatic groundwater. Drilling into the subsurface allows access to these habitats for microbial diversity study. Due to nutrient limitation, subsurface microbiomes adapt, potentially producing biotechnologically important biomolecules. *Planctomycetota*, known for possessing about 20 to 45% of protein-coding genes of unknown function, may be relevant in this context. A percolate water sample from the weathered bedrock of the Hainich Critical Zone Exploratory (CZE; Thuringia, Germany) was processed to enrich planctomycetes, leading to the isolation of an uncharacterized *Isosphaeraceae* member, strain EP7^T^. Strain EP7^T^ forms round, pink colonies, and spherical, non-motile cells that divide asymmetrically by budding. It grows between 10 and 24 °C and over a range of pH 5 to pH 10. Its genome size is 7.2 Mbp, and its DNA G + C content is 66.7%. Polyphasic characterization justifies the assignment of strain EP7^T^ to a novel species within a novel genus. We introduce the name *Kueselia aquadivae* for the novel taxon with strain EP7^T^ as the type strain of the novel species. Strain EP7^T^ represents the first *Isosphaeraceae* member isolated from vadose zone percolate water.

## Introduction

Bacteria from the phylum *Planctomycetota* are ubiquitous and play major roles in the global carbon and nitrogen cycles^1^. While being the fourth most abundant bacterial phylum in soil^2^, *Planctomycetota* members are primarily studied in aquatic habitats. They dwell mostly on all sorts of marine phototroph surfaces^3–8^, on which they can dominate the respective biofilms^9,10^ and digest complex carbon substrates^11,12^. We isolated and analyzed about a hundred members of the phylum^13,14^ from habitats as different as Monterey Bay (USA) and North Sea (Germany) kelp forests^15,16^, the macro alga *Fucus spiralis*^17^, hydrothermal environments^18–20^, marine active volcanic sites^21^, plastic particles^22,23^, wood specimens^24^, marine microbial mats^25,26^, limnic cyanobacterial blooms^27^, jellyfish^28^, marine^29^ and limnic sponges^30^, and wastewater^31^. Furthermore, autotrophic planctomycetes have been isolated, exemplified by the anammox (anaerobic ammonium oxidation) process in specialized planctomycetes (class “*Candidatus* Brocadiia”)^32^, which convert ammonium to dinitrogen gas^33^.

All planctomycetes share an unusual cell biology compared with canonical Gram-negative bacteria^34,35^. They possess unique crateriform structures, potentially involved in large polysaccharide uptake^35^. Their periplasm can be extremely enlarged, possibly for the digestion of internalized polysaccharides^35^. They divide mostly by polar budding, lacking canonical divisome proteins including the otherwise universal bacterial cell division protein FtsZ^13,36^. Identifying functional genes in planctomycetal genomes is challenging, as no other known bacterial phylum displays so many genes of unknown function^37^. Biosynthetic gene clusters (BGCs) potentially associated with secondary metabolite biosynthesis could be identified in all planctomycetal genomes sequenced thus far^13^. Indeed, *Planctomycetota* members are producers of small molecules^38–40^. These secondary metabolites probably have important bioactivities^41^. Recent examples of small molecules identified in planctomycetes include stieleriacines, potential biosurfactants^42,43^, an aromatic plant toxin^44^ and alkylresorcinols of yet unknown function^45^. Consequently, it is imperative to systematically survey additional habitats for the presence of planctomycetes and the successful cultivation of novel axenic strains is crucial to unlock their full potential.

We targeted the subsurface habitat of shallow weathered bedrock for cultivating novel members of the phylum *Planctomycetota*. Such habitats, ranging from below the soil to the groundwater, are characterized by variabilities in pore space, nutrient availability and oxygen concentrations^46,47^.

In this study, we sampled bedrock percolates, resulting from natural rainfall, employing recently developed drainage collectors within the Hainich Critical Zone Exploratory (CZE) in Thuringia, Germany and obtained strain EP7^T^ that we will describe in the context of this work. The study area stretches across a highland groundwater recharge area with different land use types^48^. Strain EP7^T^ belongs to a new taxon within the family *Isosphaeraceae*. Phylogenetically, this family is assigned to the order *Isosphaerales*, class *Planctomycetia*, phylum *Planctomycetota*. The family was first described in 2016^49^ with the type genus being described in 1987 as *Isosphaera*^50^. The current family comprises the six genera *Aquisphaera*^4^, *Isosphaera*^50^, *Paludisphaera*^49^, *Singulisphaera*^51^, *Tautonia*^52^, and *Tundrisphaera*^53^. Members of the family divide asymmetrically (by “polar budding”) and show a spherical cell shape, which is also characteristic for some planctomycetal representatives within the *Gemmataceae*^54,55^, and *Planctomycetales*^56^. Besides their spherical cell shape these orders also show ovoid, rice- or pear-shaped cells.

## Materials and methods

### Sampling and sampling site

The sampling site consists of an unmanaged mixed beech forest characterized by its natural state and only small clearings providing a unique ecological environment for studying natural seepage and percolate water. A thin soil cover (approx. 0.43 m) is located above limestone-mudstone alterations (Trochitenkalk Fm.; moTK-1;^57^ of the Upper Muschelkalk (German Triassic). In the weathered bedrock, altered, and partly secondary iron-mineral stained fractures in generally tight-matrix rock provide most important paths for percolation^57,58^.

**Fig. 1:**
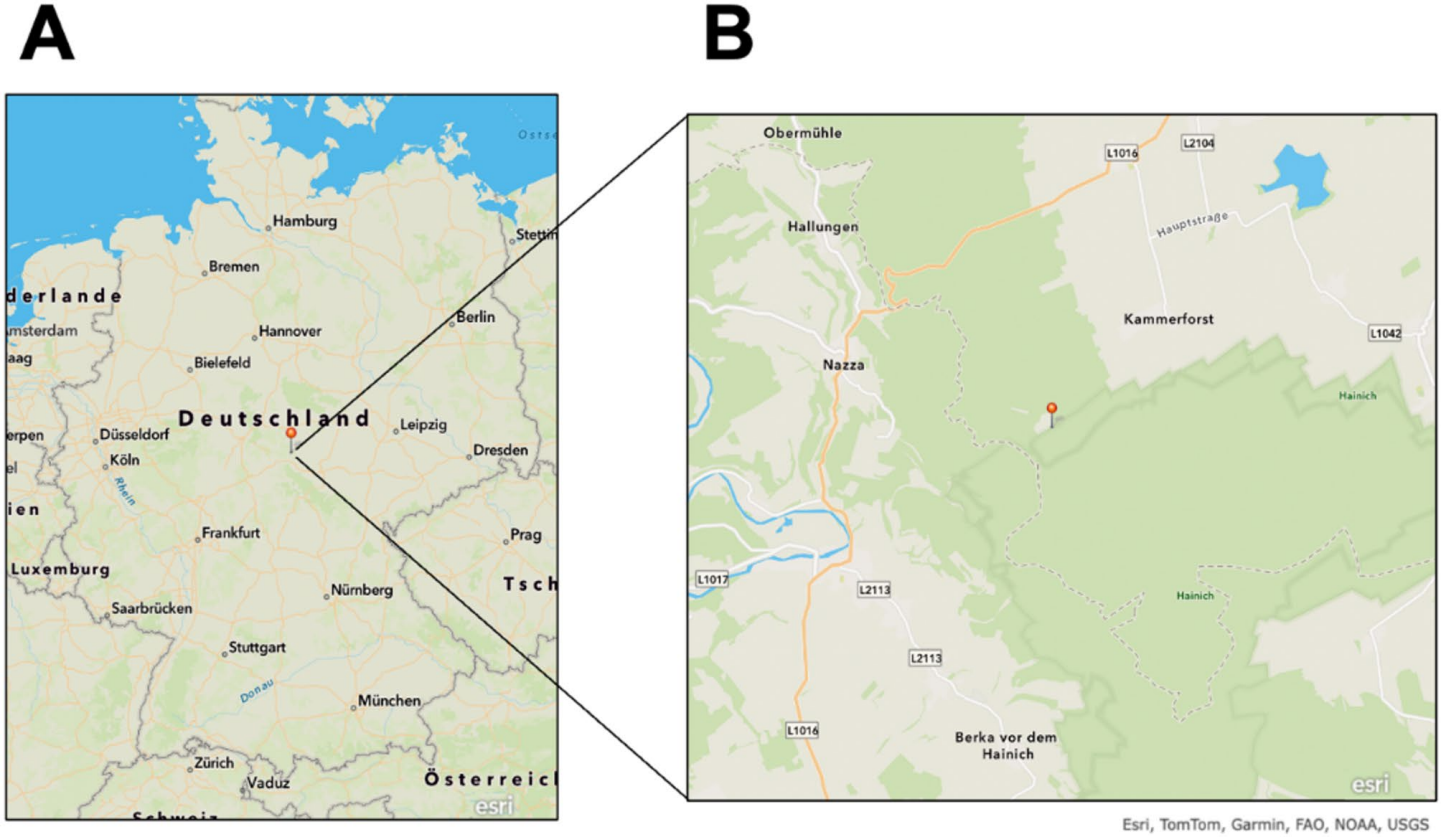
Location of the sampling site. (A) Location of the Hainich CZE in Thuringia, Germany (indicated with the pin needle). (B) Isolation of strain EP7^T^ from a sample from the Drainage Collector ADI-DC-TK-01 (TK-1) in a mixed beech forest area in the Hainich CZE, maintained by the Collaborative Research Center AquaDiva. The maps were created using the ArcGis online function MapViewer provided by Esri (https://www.arcgis.com/home/webmap/viewer.html, version 02/2024, accession date: 14/06/2024).

The sampling took place on March 11, 2020, in the Hainich CZE, Thuringia, Germany (51.10191 N, 10.397129 E). The percolate sample (HAI TK-1 11032020) originates from a drainage collector (ADI-DC-TK-01) that accesses weathered bedrock/regolith, ~ 1.40 m below the surface at a natural slope (Fig. 1). The drainage collector (DC) consists of an upward-open half-pipe (PVC-U) with an inner removable trough (same material) for cleaning. The DC was installed into a slightly inclined borehole with a diameter of approx. 140 mm to collect percolates and fracture drip water. The collection bottle (amber borosilicate glass) housed in a light-protected enclosure is connected via PUR tubing to a full-pipe section, capped outside the rock. In total, a volume of 60 mL with a pH of 7.6 was sampled after an accumulation period of 13 days. The percolate was transported in the dark at 4 °C and was quickly aliquoted in the laboratory for further processing. The percolate water was further characterized with a total organic carbon (TOC) concentration of 9.86 mg/L, dissolved organic carbon (DOC) concentration of 5.57 mg/L, particulate organic carbon (POC) concentration of 4.29 mg/L, and an electrical conductivity of 161.0 µS/cm.

### Isolation and cultivation

Strain EP7^T^ was isolated using limnic M1 medium (M1H NAG AFW, artificial freshwater (AFW) buffered with HEPES (H) and supplemented with *N*-acetylglucosamine (NAG))^31^. Limnic M1 medium plates (containing 15 g/L agar) were supplemented with 20 mg/L cycloheximide, 20 mL/L nystatin and 100 mg/L ampicillin to suppress growth of fast-growing bacteria and fungi. Plates were incubated in duplicates using 50 µL of sample and were incubated at 18 °C in the dark and under oxic conditions. Plates were regularly inspected for growth; single colonies were isolated and transferred to new media plates. The axenic status of a culture was verified by PCR-based amplification of the 16S rRNA gene using a previously published protocol^59^. Once the axenic status was confirmed, cultures were cultivated on plates and liquid limnic M1 medium without antibiotics.

### DNA extraction, genome sequencing, and genome assembly

Isolation of genomic DNA, long-read sequencing with Oxford Nanopore, genome assembly and polishing of the assembly with Illumina short reads as well as post-processing was performed as described^31^.

### Nucleotide sequence accession numbers

The 16S rRNA gene sequence of the novel isolate was deposited in the NCBI GenBank database under the accession number PP623702. The genome of strain EP7^T^ (assembly GCA_038400315.1) was deposited under the accession numbers CP151667 (chromosome), CP151668 (pEP7_01), CP151669 (pEP7_02), CP151670 (pEP7_03), and CP151671 (pEP7_04).

### Phylogenetic analysis

The 16S rRNA gene sequences of strain EP7^T^ and all characterised members of the current phylum *Planctomycetota* were aligned with ClustalW^60^. The alignment was used to calculate a 16S rRNA gene sequence similarity matrix with TaxonDC^61^. A 16S rRNA gene sequence-based maximum likelihood phylogenetic tree was calculated from the same alignment with FastTree 2.1^62^ employing the GTR + CAT model and 1000 bootstraps replications. Three 16S rRNA genes of bacterial strains from the *Planctomycetota*-*Verrucomicrobiota*-*Chlamydiota* (PVC) superphylum outside of the phylum *Planctomycetota*, namely *Opitutus terrae* (NCBI acc. no. AJ229235), *Kiritimatiella glycovorans* (acc. no. NR_146840) and *Lentisphaera araneosa* (acc. no. NR_027571), were used as outgroup. The multi-locus sequence analysis (MLSA)-based tree was constructed using autoMLST with 500 bootstrap replications^63^. The analysis was performed with the autoMLST-simplified-wrapper tool available on GitHub based on the reference genomes of all current members of the family *Isosphaeraceae*. The genomes of *Rhodopirellula baltica* SH1^T^ (GenBank acc. no. BX119912.1), *Stieleria maiorica* Mal15^T^ (acc. no. CP036264.1) and *Planctopirus limnophila* DSM 3776^T^ (acc. no. GCA_000092105.1) (belonging to the families *Pirellulaceae* or *Planctomycetaceae*) served as outgroup. Average amino acid identities (AAI) and average nucleotide identities (ANI) were calculated using the respective scripts of the enveomics collection^64^. The percentage of conserved proteins (POCP) was calculated as described^65^. The *rpoB* gene sequences encoding the β-subunit of RNA polymerase were extracted from publicly available genome annotations and sequence identities were determined as previously described^66^. The alignment and matrix calculation were performed upon extracting a ca. 1300 bp region of the *rpoB* coding sequence that would have been sequenced with the described primer set. Alignment and matrix calculation were performed with ClustalW^60^.

To evaluate the occurrence of strain EP7^T^ in local groundwater and seepage, we compared its 16S rRNA gene sequence with previously published bacterial 16S rRNA gene datasets obtained from groundwater and seepage water from the Hainich CZE^67–70^. In addition, sequences from another well (H13) was included, which now encompassed 267 groundwater and 174 seepage water samples collected between 2013 and 2019. After removing primers, we employed the R package “dada2” (v1.26)^71^ to acquire amplicon sequence variants (ASVs), followed by taxonomy assignment based on the SILVA database (v138.1)^72^. The ASVs clustering within the phylum *Planctomycetota* were pairwise aligned with strain EP7^T^ using the ‘Biostrings’ (V 2.66) package. Closely related ASVs were then compared using ClustalW alignment, and similarity values were calculated through a distance matrix, both performed with Mega software (version 11.0.13). To ascertain the phylogenetic placement of the ASVs and strain EP7^T^, an additional maximum likelihood tree based on the 16S rRNA gene sequence was constructed using Mega software. Sequence data generated in this study was submitted to the European Nucleotide Archive (ENA) with the project number PRJEB78701 under accession number ERR13457364.

### Analysis of genome-encoded features

The “Estimate Metabolism” function of anvi’o v. 8^73^ was used for the analysis of genome-encoded primary metabolic functions. The number of genes coding for putative carbohydrate-active enzymes (CAZymes) was obtained from the genome annotation provided by eggnog-mapper v.2.1.12^74^. An in silico prediction of BGCs putatively involved in the biosynthesis of secondary metabolites was carried out using antiSMASH 7^75^. The prediction was run with relaxed strictness and all extra features activated. The genome completeness was assessed with BUSCO v5.4.7^76^, while the coding density was analysed with CheckM v1.1.6^77^.

### Physiological tests

Physiological tests were performed using limnic M1 medium^13^. To determine temperature tolerance, media plates were incubated as biological duplicates with 150 µL liquid culture at 4, 10, 18, 21, 24, 28, 32, 37, and 40 °C. Subsequently, pH tolerance was analysed using 5 mL liquid cultures incubated at 21 °C in biological duplicates. The media used for cultivation were buffered with 2-(*N*-morpholino)ethanesulfonic acid (MES) for pH 5–6, with 4-(2-hydroxyethyl)-1-piperazineethanesulfonic acid (HEPES) for pH 7–8, or with *N*-cyclohexyl-2-aminoethanesulfonic acid (CHES) for pH 9–10. The tested pH values included 5.0, 6.0, 7.0, 7.5, 8.0, 9.0, and 10.0. The pH was adjusted with KOH or HCl depending on the used buffer substance. The evaluation of growth was based on visual inspection of the turbidity of the cultures. Additionally, the capability of anoxic growth was tested by inoculating strain EP7^T^ in anaerobic limnic M1 medium. The medium was anaerobized by flushing it with dinitrogen gas for two minutes until oxygen concentrations below 1 µmol/L were achieved. The inoculated vessels were then transferred into an anaerobic chamber (BugBox Ax, Baker Company) to avoid oxygen influx and frequently checked for growth by measuring the OD_600_. Furthermore, strain EP7^T^ was inoculated into anaerobic limnic M1 medium separately supplemented with either 10 mM sodium fumarate, 10 mM sodium sulphate, or 3 mM sodium nitrate as alternative electron acceptors, to test for anaerobic respiration. The culture was incubated at 18 °C. Oxygen concentrations were measured using the fiber optic oxygen transmitter Microx 4 connected to a PSt 7 oxygen sensor (PreSens Precision Sensing) to ensure oxygen- free growth conditions (< 0.20 µM).

Strain EP7^T^ was compared, whenever possible, with its closest relative *Tundrisphaera lichenicola* P12^T^ and related type strains from the family *Isosphaeraceae*.

### Light microscopy and cell size determination

Image acquisition and cell size determination were conducted as previously described^31^. Briefly, three replicates were grown in limnic M1 medium at 18 °C until the half-maximal OD_600_ was reached. On the day of imaging, cells were immobilized on a 1% (w/v) agarose cushion (dissolved in limnic M1 medium). Images were taken with a Nikon Ti2 inverted microscope equipped with a Nikon Plan Apo λ 100x immersion oil objective (with a phase ring for PhC images), a Nikon DS-Ri2 camera, and the NIS-Elements software (version 5.30). Cells (three replicates with 199 cells each) were analyzed with BacStalk (Version 1.8)^78^ and the resulting data was plotted in SuperPlotsofData^79^.

Time-lapse experiments were performed with the same microscope used for cell size determination. For imaging, strain EP7^T^ was cultivated at 18 °C and cells were immobilized on a 1% (w/v) agarose cushion (dissolved in limnic M1 medium). The sample was sealed with VLAP (33% vaseline, 33% lanoline, 33% paraffin, w/w) to prevent evaporation during imaging. Images were taken every 15 min at multiple locations. For visualization purpose only, the brightness and contrast were adjusted in FIJI^80^ and scale bars were added.

### Super-resolution microscopy

To investigate the ability of the strain to take up complex polysaccharides and to analyze the cell plan of strain EP7^T^, super-resolution microscopy experiments were performed with a Zeiss Elyra7 Lattice SIM Microscope equipped with the respective filters/lasers: LP570/561 nm (used for Synaptored), BP420-480 + LP750/405 nm (used for DAPI), and BP525/50/488 nm (used for fluorescein isothiocyanate (FITC)-labelled dextran) and a Plan-Apochromat 63x/1.4 Oil objective. Structured illumination microscopy (SIM) images were reconstructed and aligned using the in-built function of the ZenBlack software (Zeiss). The strain was cultivated at 18 °C and on the day of imaging 500 µL of culture were subjected to 15 min of staining with 5 µL FITC-labelled dextran (Sigma-Aldrich, average molecular weight 40,000, final concentration: 5 µg/mL). After 5 min of dextran feeding, 3 µL 4′,6-diamidino-2-phenylindole (DAPI; 500 mg/ml) and 1 µL Synaptored (3 mg/ml) were added and staining was continued for the remaining 10 min. Afterwards, the culture was washed two times with centrifugation for three minutes at 1,000 x g, the resulting pellet was resuspended in limnic M1 medium. After the washing steps, cells were resuspended in 300 mL mounting medium (20 mM Tris pH 8.0, 0.5% (w/v) *N*-propyl gallate, 80% (v/v) glycerol). Cells were immobilized on a 1% (w/v) agarose cushion (dissolved in limnic M1 medium) and the mount was sealed with VLAP. For visualization purposes, the brightness and contrast were adjusted in FIJI and scale bars were added.

## Results and discussion

### Isolation of strain EP7^T^

To date, no representative of the family *Isosphaeraceae* has been successfully isolated from the undisturbed subsurface and is available as an axenic culture. The obtained samples were used as inoculum and incubated on limnic M1 medium agar plates supplemented with a mixture of carbon sources and antibiotics suitable to enrich planctomycetes. Since these bacteria tend to have low growth rates and often produce carotenoids as pigmenting compounds, we prioritized colonies that were pink to red in color and only appeared after several weeks of incubation. Selected colonies were passaged three times on agar plates to obtain an axenic culture, which was validated by 16S rRNA gene amplification and sequencing. Strain EP7^T^ grows on glucose as carbon source, supplemented with NAG as nitrogen- and additional carbon source.

### Phylogenetic inference

**Fig. 2:**
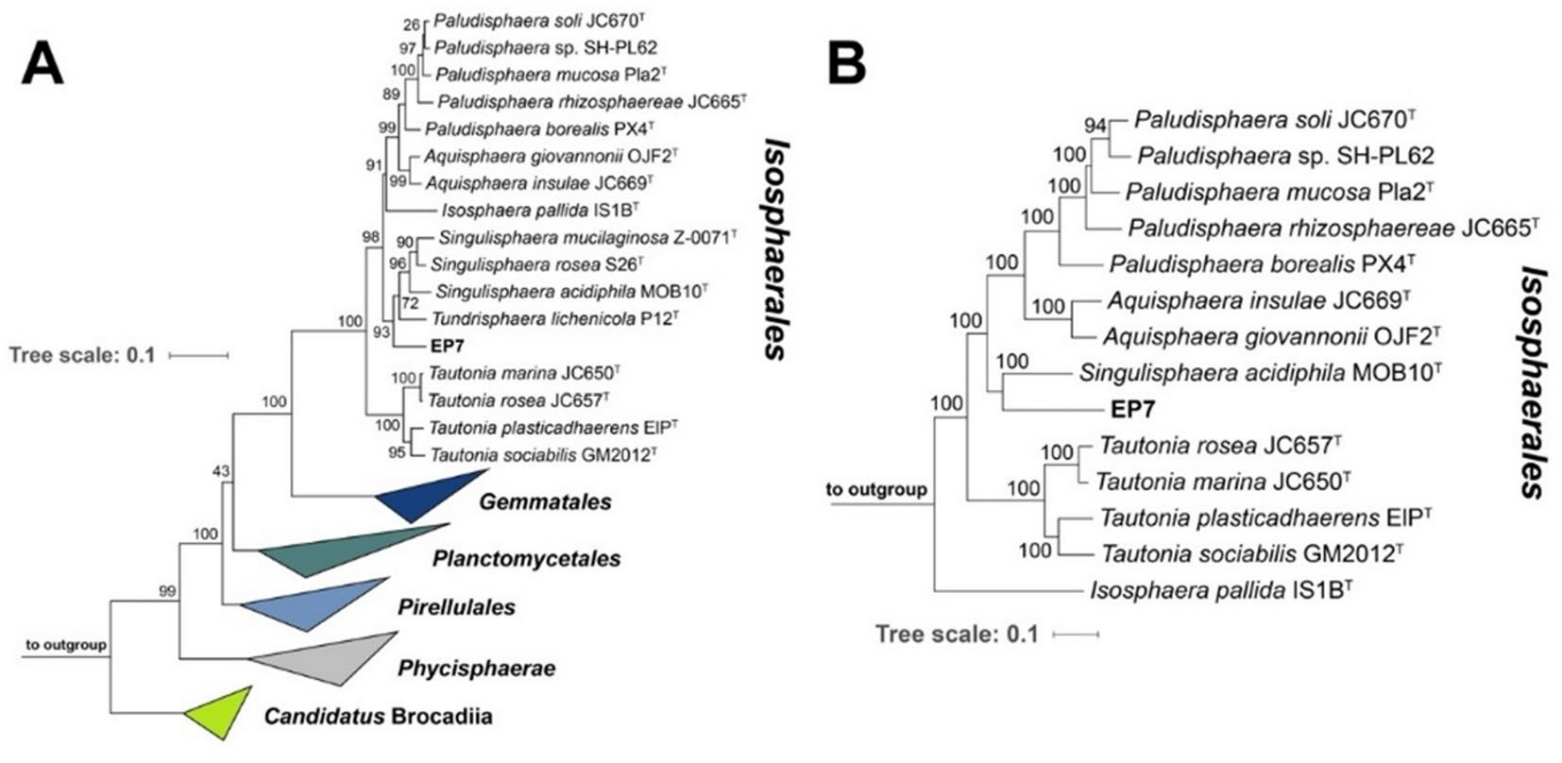
Maximum likelihood phylogenetic trees displaying the phylogenetic position of the novel strain isolated from the undisturbed subsurface. Maximum likelihood phylogenetic trees based on 16S rRNA gene sequences (A) and MLSA (B) were computed based on the current members of the phylum *Planctomycetota* (A) or the current members of the family *Isosphaeraceae* (B) (+ outgroups as given in the Material and methods section). Bootstrap values after 1000 re-sampling (A) or 500 re-samplings (B) are given at the nodes (in %). The trees were visualized using iTOL v6. The scale bar indicates the number of substitutions per nucleotide (A) or amino acid position (B).

**Fig. 3:**
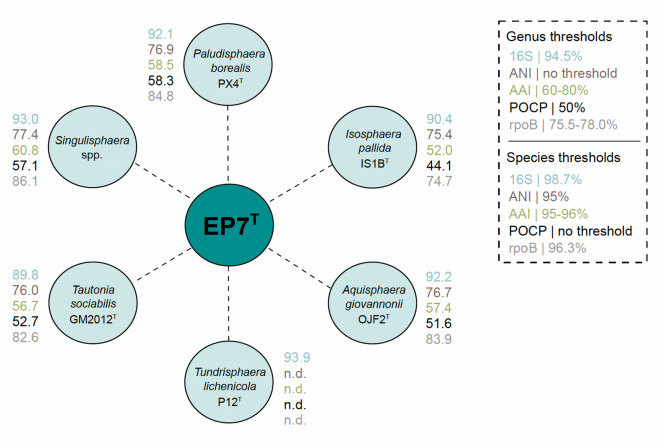
Analysis of phylogenetic markers. Strain EP7^T^ and its current closest relatives are displayed with the values of respective phylogenetic markers including 16S rRNA gene sequence similarity (16S), average amino acid identity (AAI), average nucleotide identity (ANI), percentage of conserved proteins (POCP) and similarity of a 1300 bp partial *rpoB* sequence. The genus/species threshold for each genetic marker is shown in the upper right corner. n.d. not determined (no genome sequence available). *The genus threshold for *rpoB* was determined for members of the families *Planctomycetaceae* and *Pirellulaceae* and might not be applicable for the other orders in the class *Planctomycetia*.

A Blastn analysis of the initially obtained partial 16S rRNA gene sequence of strain EP7^T^ indicates a close relationship to characterized members of the family *Isosphaeraceae*. The highest sequence similarity of 93.9% was obtained for comparison with *T. lichenicola* P12^T^ that was isolated from the upper oxic layer of lichen-dominated peatland in Russia^53^. The phylogenetic position of strain EP7^T^ in the family *Isosphaeraceae* (the currently sole family in the order *Isosphaerales*) was confirmed using maximum likelihood phylogenetic trees based on 16S rRNA gene sequences and MLSA (Fig. 2). Since the genome of *T. lichenicola* P12^T^ has not been sequenced yet, our isolate clustered with *Singulisphaera acidiphila* MOB10^T^ in the MLSA-based tree. *S. acidiphila* was also identified as a close relative apart from *T. lichenicola* P12^T^ in the 16S rRNA gene sequence-based tree along with two other *Singulisphaera* species for which the type strains currently lack genome sequencing data^51,81,82^. Both tree positions display good overall bootstrap support (93–100%). To further analyse the phylogenetic position of strain EP7^T^, five phylogenetic markers were evaluated: 16S rRNA gene sequence similarity, AAI, ANI, POCP and similarity of a ca. 1300 bp partial sequence of the gene *rpoB* coding for the β-subunit of RNA polymerase^66^ (Fig. 3).

A maximal ANI value of 77.4% (species threshold: 95%^83^ and a 16S rRNA gene sequence similarity far below the species threshold of 98.7% between strain EP7^T^ and the current members of the six genera in the family showed that the novel isolate does not belong to a described species. The above-mentioned maximal 16S rRNA gene sequence similarity to *T. lichenicola* P12^T^ (93.9%) turned out to fall below the genus threshold of 94.5% pointing towards a relationship on the level of separate genera. The analysis of whole genome-based phylogenetic markers had to exclude the current closest relative due to lack of a sequenced genome. The AAI values obtained during comparison of our isolate with strains with a sequenced genome supported the notion that strain EP7^T^ belongs to a separate genus. However, the POCP and *rpoB* values fell above the respective genus thresholds of 50% and 75.5–78%, respectively^65,84^. The *rpoB* similarity values have only limited validity since the empirically determined threshold values are so far only applicable for the orders *Planctomycetales* and *Pirellulales* and were analysed for the sake of consistency with previous strain description manuscripts. In general, the *rpoB* gene is known to be horizontally transferred between bacterial species^85,86^. However, horizontal gene transfer of *rpoB* within the phylum *Planctomycetota*^87^ has never been described. Horizontal gene transfer (HGT) often occurs under highly selective (laboratory) settings, e.g. use of (multiple) antibiotics^88,89^, but only under specific conditions successfully in natural habitats^90^. Therefore, the unexpected high similarity of the *rpoB* gene might not be the result of HGT, but the relict of still improvable *rpoB* similarity thresholds within the order *Isosphaerales*. Furthermore, as *rpoB* is a critical, highly conserved component of the transcriptional machinery of bacteria, by encoding the β-subunit of RNA polymerase, a successful transfer under natural conditions is highly unlikely and a reason for its use in phylogenetic studies.

The obtained POCP values are unexpectedly high and in conflict with the AAI values that fell either below or at the lower boundary of the genus threshold range (Fig. 3). Taken together, the phylogenetic inference suggests the type strain of *T. lichenicola* as the current closest neighbour of strain EP7^T^. The similarity of the 16S rRNA gene sequences is in line with the delineation of the strain from the genus *Tundrisphaera*. Hence, we will assign the strain to a novel species of a novel genus if this notion is supported by significant differences obtained during the polyphasic characterization.

### Comparison of genomic features

Since the genome of the type strain of the most closely related species *T. lichenicola* has not yet been sequenced, we compared the genomic features of strain EP7^T^ against the next relative with a sequenced genome, which is *S. acidiphila* MOB10^T^ (Table 1).

**Table 1:** Comparison of genomic features of strain EP7^T^ and *Singulisphaera acidiphila* MOB10^T^. Abbreviations: PKS: polyketide synthase, NRPS: non-ribosomal peptide synthetase, RiPP: ribosomally synthesized and post-translationally modified peptides, NAPAA: non-alpha poly-amino acid.

Characteristics	EP7^T^	*Singulisphaera acidiphila* MOB10^T^^51^
Genome size (bp)	7,199,273	9,755,686
Plasmids	4	3
DNA G + C content (%)	66.7	62.2
Coding density (%)	86.3	83.5
Completeness (Busco) (%)	98.4	99.3
Genes (total)	5,685	7,327
Genes per Mbp	790	751
Protein-coding genes	5,563	7,163
Protein-coding genes per Mbp	773	734
Hypothetical proteins	1,534	1,841
Hypothetical proteins (%)	27.6	25.7
rRNAs (5S, 16S, 23S)	4, 4, 4	8, 8, 8
tRNAs	58	71
Carbohydrate-active enzymes		
Glycoside hydrolases	19	35
Glycosyl transferases	24	32
Polysaccharide lyases	1	2
Carbohydrate esterases	0	2
Carbohydrate-bind. modules	3	5
Total	47	76
CAZyme genes per Mbp	6.5	7.8
AntiSMASH Biosynthetic gene clusters		
Terpenoid	3	3
Type I PKS	3	2
Type III PKS	1	0
Mixed type I PKS-NRPS	0	2
NRPS-like	0	1
RiPP-like	0	1
Phenazine	0	1
NAPAA	0	1
Total	7	11
BGCs per Mbp	1.0	1.1

The genome of strain EP7^T^ has a size a 7.20 Mbp and a DNA G + C content of 66.7%. Its genome is more than 2.5 Mbp smaller than that of *S. acidiphila* MOB10^T^ and differs in the DNA G + C content by more than 5 percentage points. The presence of plasmids (with sizes of 70, 58, 41 and 34 kbp) in the novel isolate was expected since all characterized members of the family have at least one and a current maximum of five plasmids^18,91^. The difference in the genome size is reflected by the numbers of protein-coding genes, tRNA and rRNA genes (Table 1). Strain EP7^T^ has four sets of rRNA genes (5S, 16S, 23S), *S. acidiphila* has even eight sets. A slightly lower coding density in *S. acidiphila* is in line with a 5% lower number of protein-coding genes per Mbp compared to strain EP7^T^. The relative number of genes encoding hypothetical proteins falls between 26 and 28% in both analyzed genomes and is, to our surprise, lower in the strain with the larger genome. Members of the phylum *Planctomycetota* are known to have high numbers of genes with an unknown function, with typical relative numbers between 20 and 45% depending on the size of the genome and the tool used for automated genome annotation.

Comparison of strain EP7^T^ with the local groundwater and seepage microbiomes identified three closely related ASVs: ASV 1263, ASV 8215, and ASV 69341. Similarity values of strain EP7^T^ and these ASVs indicated affiliations to other genera, with similarities of 91.6% (ASV 1263), 91.9% (ASV 8215), and 93.5% (ASV 69341) over the length of ca. 400 bps of the ASVs, respectively. Figure S1 shows the phylogenetic position of strain EP7^T^ and the ASVs, with ASV 69341 related to *T. lichenicola* P12^T^ and ASVs 8215 and 1263 related to *Singulisphaera* spp., in a 16S rRNA gene sequence-based phylogenetic tree, including current members of the phylum *Planctomycetota*.

### Genome-based analysis of primary and secondary metabolic functions

The determined numbers of carbohydrate-active enzymes (CAZymes) and biosynthetic gene clusters (BGCs) related to secondary metabolite biosynthesis turned out to reflect the difference in the genome size (Table 1). The genomes of strain EP7^T^ and *S. acidiphila* MOB10^T^ harbor six to eight putative CAZyme-encoding genes and one BGC per Mbp. While the CAZyme class distribution is similar, with most putative members falling in the classes of glycoside hydrolases and glycosyltransferases, the BGC diversity differs significantly. The predicted BGCs in strain EP7^T^ are restricted to genes coding for proteins putatively involved in the biosynthesis of terpenoid- and polyketide-derived products, whereas putative biosynthetic proteins for additional compound classes (phenazines, non-ribosomal peptides, non-alpha poly-amino acids) were detected in the genome of *S. acidiphila* MOB10^T^. The genome mining suggests *S. acidiphila* as the more talented producer of biosynthetic compounds. Whether the “reduced” genome of strain EP7^T^ is related to the oligotrophic habitat from which it was isolated remains speculative at this stage and requires genome information of additional isolates.

The “Estimate Metabolism” function of anvi’o 8 was used to predict the completeness of primary metabolic pathways based on the genomes of strain EP7^T^ and *S. acidiphila* MOB10^T^. The analysis points towards a canonical central carbon metabolism of a typical aerobic and heterotrophic bacterium. Glycolysis (Embden-Meyerhof pathway), gluconeogenesis, pentose phosphate pathway, tricarboxylic acid cycle and electron transport chain appear functional since all required genes were found in the genomes. The same is true for anabolic pathways for biosynthesis of fatty acids, amino acids, nucleotides and vitamins or cofactors (biotin, tetrahydrofolate, riboflavin, nicotinamide, menaquinone, etc.). Both strains use the non-mevalonate pathway for the synthesis of isoprene units and are predicted to be resistant to β-lactam antibiotics. They also have most of the catabolic pathways in common, for example the glycine cleavage system, proline degradation and galactose degradation via the Leloir pathway.

### Physiological analyses and in situ occurrence

**Fig. 4:**
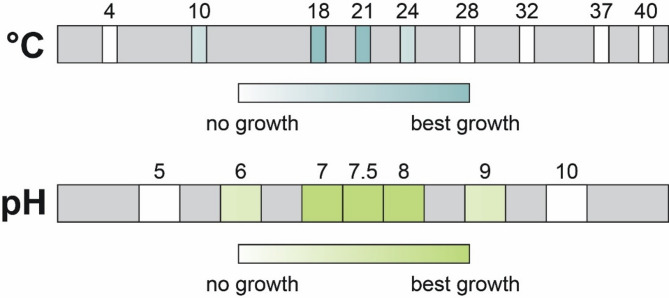
Determination of temperature and pH range and optimum. Strain EP7^T^ grows over a range of 10 to 24 °C with an optimum between 18 to 21 °C and between pH 6.0 and 9.0 with an optimum at 7.0 to 8.0.

To determine the optimal growth conditions, strain EP7^T^ was inoculated on limnic M1 medium agar plates and duplicates of the inoculum were placed at various temperatures under oxic conditions. Based on the appearance of single colonies and their size, strain EP7^T^ was confirmed to grow over a temperature range of 10 to 24 °C. No growth was observed below or above these temperatures, the optimal growth temperature was found to fall between 18 and 21 °C (Table 2; Fig. 4). Notably, strain EP7^T^ has a more restricted range of growth temperatures compared to its closest relative *T. lichenicola* P12^T^. *T. lichenicola* grows between 4 and 28 °C, with an optimum temperature range of 15–22 °C^53^. The three *Singulisphaera* species grow over a higher temperature range of 4–30 (or 33) °C with optimal growth between 26 and 28 °C^51,81,82^.

**Table 2:** Physiological and phenotypic features of EP7^T^ in comparison to its closest relatives *Tundrisphaera lichenicola* PT12^T^ and *Singulisphaera acidiphila* MOB10^T^, n.o.: not observed.

Characteristics	EP7^T^	*Tundrisphaera lichenicola* P12^T^^53^	*Singulisphaera acidiphila* MOB10^T^^51^
Sampling location	Hainich CZE, Thuringia (Germany)	Nadym Region, Yamalo-Nenets Autonomous Okrug (Russia)	Obukhovskoe, Yaroslavl Region (Russia)
Sampled material	Percolate of drainage collector	Oxic layer of peat	Acidic *Sphagnum* Peat (pH 4.2)
Temperature range (optimum) (°C)	10–25 (18–21)	4–28 (15–22)	4–33 (20–26)
pH Range (optimum)	6.0–9.0 (7.5)	4.5–6.8 (5.5–6.0)	4.2–7.5 (5.0–6.2)
Relation to oxygen	Facultatively anaerobic	Aerobic	Aerobic
Cell shape	Spherical	Spherical	Spherical
Pigmentation	Light pink	Pink	Colorless (opaque)
Motility	Non-motile	Non-motile	Non-motile
Stalks	n.o.	Holdfast-like appendages	Absent
Aggregates	Forms small, microscopic aggregates	Forms small chains	Forms shapeless aggregates
Cell division mode	Asymmetric cell division (“polar” budding)	Asymmetric cell division (“polar” budding)	Asymmetric cell division (“polar” budding)

Strain EP7^T^ showed no growth below pH 6.0 and above pH 9.0. Optimal growth could be observed between pH 7.0 and 8.0 (Fig. 4). Hence, strain EP7^T^ is neutrophilic in contrast to the type strain of *T. lichenicola*, which grows over a pH range of 4.5 to 6.8 (slightly acidic) with an optimum pH range between 5.5 and 6.0^53^. The optima of both strains reflect the environment from which they were isolated. *T. lichenicola* P12^T^ seems to be adapted to slightly acidic pH values, which is in accordance with the acidic pH values reported for tundra peat soil^92^. Strain EP7^T^ appears to be well adapted to neutral/slightly alkaline pH values, consistent with the conditions observed during the sampling campaign, where a pH of 7.6 was recorded. Typical pH values of the groundwater in the Hainich CZE, Thuringia, Germany, are around 7.2, with occasional peaks up to pH 8.0^48^. This consistency suggests that strain EP7^T^ is well adjusted to the local pH milieu.

Strain EP7^T^ is capable to grow under oxic and anoxic conditions and thereby is regarded a facultative anaerobe. The next relatives *T. lichenicola* and *S. acidiphila*, however, are described as obligate aerobic planctomycetes^51,53^, which might show an adaption of strain EP7^T^ to its subsurface habitat. Anoxic growth of strain EP7^T^ was observed in limnic M1 medium supplemented with 10 mM sodium fumarate after 2 weeks of inoculation. No significant growth was observed in limnic M1 and limnic M1 medium containing 10 mM sodium sulphate or 3 mM sodium nitrate, respectively. The ability to grow under micro-oxic conditions has previously been documented in members of the family *Isosphaeraceae*, e.g. within the genus *Paludisphaera*^49^. Compared to *Isosphaera pallida* IS1B^T^, the type species of the type genus in the family *Isosphaeraceae*, the aerobic lifestyle and optimum pH (*I. pallida*: pH 7.8–8.8) are similar^50^. However, strain IS1B^T^ has a motile phototactic lifestyle and can tolerate much higher temperatures (growth from 34 to 55 °C), which is coherent to its isolation from a hot spring^50^.

Another member of the *Isosphaeraceae* family, *Tautonia sociabilis* GM2012^T^, was isolated from a microbial biofilm located in a thermal water stream (pH 8.0, 42 °C) in a gold mine in South Africa^52^. Similar to strain EP7^T^, strain GM2012^T^ has an aerobic lifestyle, the pH optimum of strain GM2012^T^ is similar (pH 7.5–7.7) to EP7^T^ and, similar to *I. pallida* IS1B^T^, strain GM2012^T^ can tolerate higher temperatures of up to 46 °C^50,52^. Strain GM2012^T^ was also isolated from a subsurface habitat; however, this habitat was built in 1957 to especially mine gold ores^93^ and therefore is heavily exposed to anthropogenic influences resulting in a non-natural habitat in which microbial communities are altered due to mining-impacted substrates^94^. Strain EP7^T^ originating from a drainage collector, that was placed into weathered but intact regolith only collecting percolates and fracture drip water without any exposed surfaces, therefore is the first member of the family *Isosphaeraceae* from an undisturbed, pristine subsurface habitat.

To further evaluate the occurrence of strain EP7^T^ in the subsurface, we screened available 16S rRNA gene data sets from time series of seepage, drainage and groundwater bacteria obtained from the Hainich CZE for close relatives. In general, none of them reached high relative abundances, with ASV 1263 and ASV 8215 being the highest in seepage with a relative abundance between 0.01 and 2.8%. ASV 69341 was found only once in a very shallow groundwater well, located in the recharge area, in a low relative abundance of 0.005%. The results indicate that strain EP7^T^ is not dominant in the local groundwater or seepage microbiomes. Although the related ASVs can occur in groundwater, they do not thrive there. The rare presence and low relative abundance of closely related ASVs evidence the difficulty of isolating strain EP7^T^.

### Morphological characterization

Having found the most suited cultivation conditions for strain EP7^T^, we conducted microscopic analyses to learn more about the cell shape, cell size, and mode of cell division. Colonies of strain EP7^T^ display a light pinkish color with a spherical form and entire margins (Table 2). Colonies show a convex elevation, and a shiny appearance measuring 0.2–1.8 mm in diameter. Despite shaking of the liquid cultures, a light pinkish sedimented pellet was formed after a designated time of incubation, which might be a result of the ability to from loose aggregates.

**Fig. 5:**
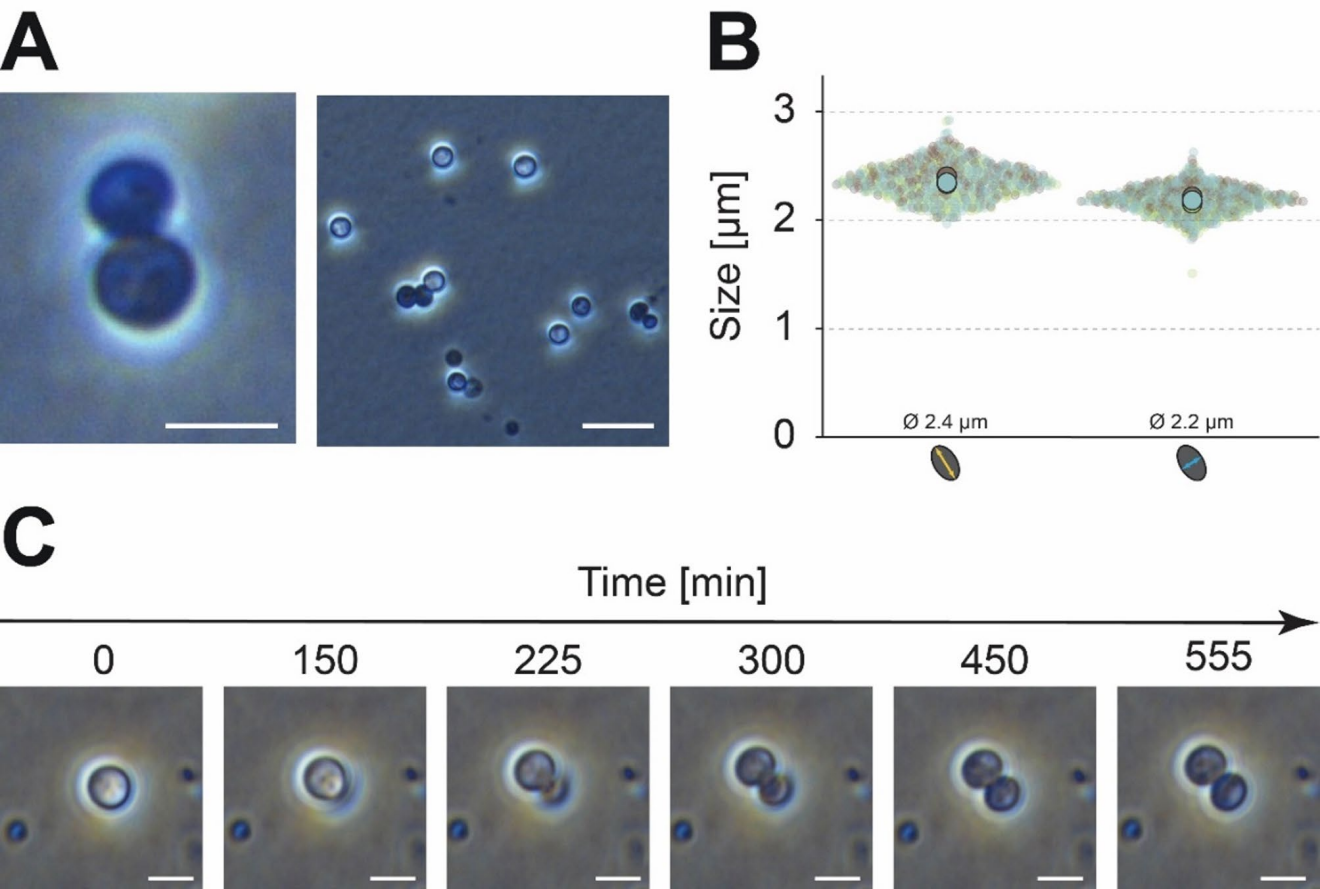
Morphological features and cell division of strain EP7^T^. (A) Phase contrast microscopy reveals cells of strain EP7^T^ to be spherical and to appear as single cells, pairs of two, or small chains. The scale bar represents 2 μm. (B) Cells of strain EP7^T^ have a mean length of 2.4 μm and a mean width of 2.2 μm representing an almost spherical shape. (C) Phase contrast time-lapse microscopy of strain EP7^T^ showing cell division via asymmetric cell division (“polar budding"). Scale bars represent 2 μm.

Microscopic analyses revealed that cells of strain EP7^T^ are arranged in loose aggregates, pairs, or single cells (Table 2; Fig. 5A). Cells have an average size of 2.4 μm and 2.2 μm in length and width, respectively, their cell shape is almost spherical (Fig. 5B). As seen in all members of the class *Planctomycetia*^1,34^, cells of strain EP7^T^ divide via asymmetric cell division (“polar budding"). Due to their spherical shape a cell pole cannot be attributed to either of the cell sides. Nevertheless, cell division starts, typical for the planctomycetal budding process, with the formation of a small daughter cell (bud) on one side of the cell. The daughter cell then increases in size over time until reaching the size of the mother cell (Fig. 5C) and final septation occurs.

Compared to *I. pallida*, the spherical shape and asymmetric cell division seem to be common features^50^. However, *I. pallida* IS1B^T^ forms larger cells (2.5–3.0 μm).

Similar to *T. sociabilis* GM2012^T^, strain EP7^T^ has non-motile cells, divides by asymmetric division (“polar” budding), and can be found as single cells or small microscopic aggregates^52^.

### Strain EP7^T^ takes up complex polysaccharides into the periplasmic space

**Fig. 6:**
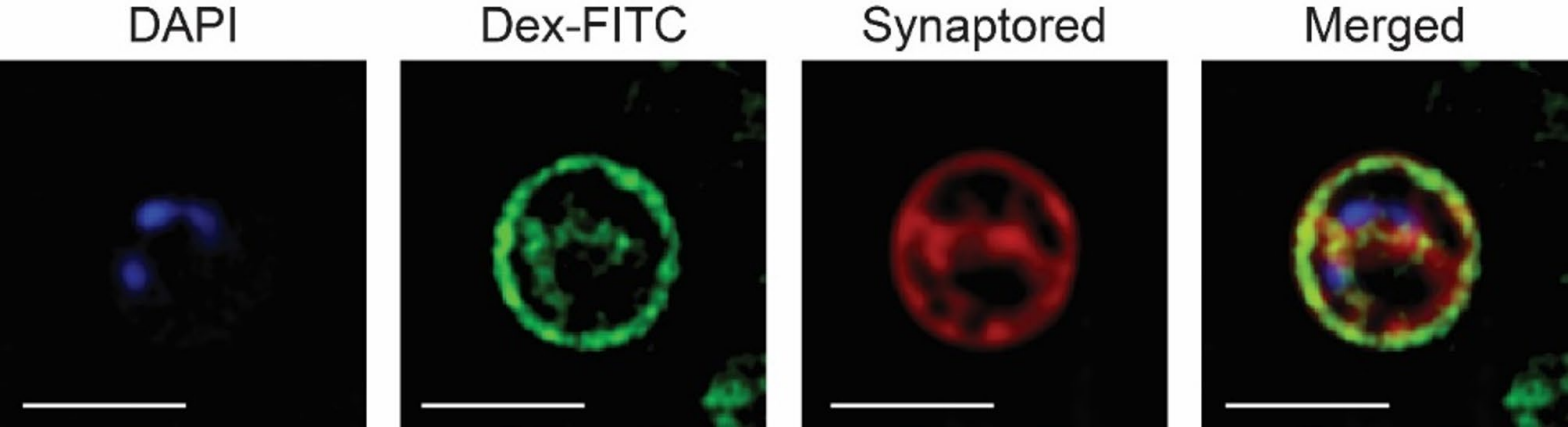
Strain EP7^T^ takes up the FITC-labelled model polysaccharide dextran. Structured illumination microscopy of strain EP7^T^ cells incubated with DAPI (DNA stain), FITC-labelled dextran, and Synaptored (membrane stain). Fluorescence signals of the FITC-labelled dextran (green) co-localize with the fluorescence signal of the membrane stain (red) indicating an uptake of the polysaccharide into the periplasmic space. The DNA appears as condensed foci and is located in the cytoplasm next to the cytoplasmic membrane and periplasmic invaginations. Scale bars represent 2 μm.

According to a previous study^34^, model strains of the phylum *Planctomycetota* are able to take up high-molecular-weight carbon sources, for example the model polysaccharide dextran, and possess an enlarged periplasmic space apparently involved in the temporary storage and subsequent degradation of such polymers. As it is known that plant-derived polysaccharides, like starch and cellulose, can enter the subsurface and can even reach groundwater aquifers by percolating through the subsurface^48,95,96^, we tested if strain EP7^T^ is able to take up such polysaccharides. To this end, we incubated cells of strain EP7^T^ with FITC-labelled dextran and simultaneously stained the membrane with Synaptored and the DNA with DAPI. A subsequent SIM analysis revealed that cells of strain EP7^T^, similar to other members of the phylum^34^, possess a condensed nucleoid and a cytoplasmic membrane which can be invaginated into the cytoplasm. Sometimes it traverses through the cytoplasm until connecting with the cytoplasmic membrane on the other side of the cell creating a periplasmic space spanning through the cytoplasm (Fig. 6). Furthermore, and in accordance with a previous publication^34^, the fluorescence signal of the FITC-labelled polysaccharide dextran could be detected inside the area stained by the membrane dye, which can be attributed to the periplasmic space of the cell (Fig. 6). This suggests that the FITC-labelled dextran was taken up by the cell and transported into the periplasm. However, the exact mechanism involved in the uptake of dextran/polysaccharides in the phylum *Planctomycetota* remains to be elucidated.

## Conclusion

Strain EP7^T^ represents the first member of the family *Isosphaeraceae* isolated from water percolating through the weathered bedrock under vadose conditions. Based on the analyzed phylogenetic, genomic, physiological, and morphological characteristics, the strain belongs to the phylum *Planctomycetota* and should be delineated from previously described genera in the family *Isosphaeraceae*. In honor of the spokesperson of the CRC AquaDiva, Kirsten Küsel, and the isolation of the strain as part of the AquaDiva project, we propose the name *Kueselia aquadivae* gen. nov., sp. nov. for the novel taxon.

### Description of *Kueselia* gen. nov.

*Kueselia* (Kue.se’li.a. N.L. fem. n. *Kueselia*; a bacterium named after Kirsten Küsel for her outstanding work in the field of geomicrobiology).

Gram-negative. Species belonging to this genus are facultatively anaerobic, heterotrophic, mesophilic, and neutrophilic. Cells are (almost) spherical and divide by asymmetric cell division (“polar budding"). Colonies show a color of light pink and a circular form. The DNA G + C content is approximately 67%. The genus belongs to the family *Isosphaeraceae*, order *Isosphaerales*, class *Planctomycetia*, phylum *Planctomycetota*. The type species is *Kueselia aquadivae*.

### Description of *Kueselia aquadivae* sp. nov.

*Kueselia aquadivae* (a.qua.di’vae. N.L. gen. n. *aquadivae*, of the AquaDiva project).

Cells have a size of approximately 2.4 μm in length and 2.2 μm in width, resulting in an almost spherical cell shape. Cells form aggregates. Growth of the type strain is observed between temperatures of 10–24 °C (optimum range 18–21 °C) and a pH of 6.0–9.0 (optimum range 7.0–8.0). The strain internalizes the model polysaccharide dextran. The type strain genome has a total size of 7,199,272 bp (chromosome and four plasmids) and a DNA G + C content of 66.7%. The type strain is EP7^T^ and was isolated from water percolating through the aeration zone with a drainage collector in the Hainich CZE, Thuringia, Germany installed by the Collaborative Research Center (CRC) 1076 AquaDiva. The strain was deposited in the Spanish Type Culture Collection (CECT) and the Jena Microbial Resource Collection (JMRC) under the accession numbers CECT 30426^T^ and STH00993^T^, respectively.

## Data Availability

The datasets generated and/or analysed during the current study are available from the corresponding author on reasonable request. 16S rRNA gene sequence and the genomic sequence generated in this study are available in the NCBI GenBank database under the accession numbers PP623702 (16S rRNA gene sequence), CP151667 (chromosome), CP151668 (pEP7_01), CP151669 (pEP7_02), CP151670 (pEP7_03), and CP151671 (pEP7_04). Amplicon sequencing data analysed in this study is available in the European Nucleotide Archive under the project number PRJEB78701 (accession number ERR13457364).
